# Aspergillus aortitis in a patient presenting with bilateral acute lower limb ischemia

**DOI:** 10.1093/jscr/rjaf418

**Published:** 2025-06-16

**Authors:** Eslam Metwalli, Richard Warwick, Julien Al Shakarchi

**Affiliations:** Vascular Surgery Department, Worcestershire Acute Hospitals NHS Trust, Charles Hastings Way, Worcester WR5 1DD, Worcestershire, United Kingdom; Cardiothoracic Surgery Department, Royal Stoke University Hospital, Newcastle Road, Stoke-on-Trent ST4 6QG, Staffordshire, United Kingdom; Vascular Surgery Department, Worcestershire Acute Hospitals NHS Trust, Charles Hastings Way, Worcester WR5 1DD, Worcestershire, United Kingdom

**Keywords:** *aspergillus niger*, aortitis, acute lower limb ischemia, cardiac surgery, aortic valve replacement

## Abstract

Aspergillus niger aortitis is a rare but life-threatening fungal infection, typically affecting immunocompromised individuals. We present a unique case of a patient presenting with bilateral acute lower limb ischemia and a recent history of aortic valve repair. Despite the absence of typical infectious symptoms, further investigation revealed an infected floating thrombus in the ascending aorta. Subsequent surgical intervention and pathological analysis confirmed the presence of Aspergillus niger, a common environmental Mold, as the causative agent of aortitis. Our case highlights the challenges of diagnosing and managing Aspergillus niger aortitis, as symptoms can be subtle, and routine laboratory tests may not always detect the infection. Previous cardiac surgery is recognized as a significant risk factor for developing Aspergillus niger aortitis. This case report underscores the importance of considering Aspergillus niger aortitis in the differential diagnosis of patients with a history of cardiac surgery who present with unexplained embolic events, such as acute limb ischemia. Immediate recognition, decisive surgical intervention, and the initiation of appropriate antifungal therapy are imperative for maximizing patient outcomes in this complex condition.

## Introduction

Aspergillus is a ubiquitous mold encountered in various environments [1]. While invasive aspergillosis primarily affects immunocompromised individuals, it can also occur after cardiac surgery, even in patients with otherwise intact immune systems. Post-cardiac surgery Aspergillus niger aortitis, characterized by fungal infection of the aorta, was first described in 1960 and is associated with significant mortality [2].

This report presents a case of Aspergillus niger aortitis in a previously healthy man who presented with bilateral acute lower limb ischemia. Notably, his only significant risk factor was a recent tissue aortic valve replacement performed 3 months prior to the onset of symptoms. This case highlights the importance of considering Aspergillus niger aortitis in the differential diagnosis of patients with recent cardiac surgery, especially those presenting with unexplained embolic phenomena. Early recognition and prompt initiation of appropriate antifungal therapy are crucial for improving outcomes in this challenging condition.

## Case history

A 74-year-old patient presented to the emergency department with bilateral lower limb pain. He woke up with acute pain affecting both legs, more so on the left side. The pain worsened with walking and did not respond to simple analgesia. He also reported a transient unilateral loss of vision that lasted a few seconds, which had occurred 3–4 weeks earlier and remained unexplained.

His past medical history included gout, hypertension, and a tissue valve replacement for severe aortic regurgitation, performed 3 months prior to this presentation. Initial blood work revealed elevated white blood cells (12.5) and low sodium (131), but was otherwise unremarkable.

On examination, his vital signs were within normal limits. Both lower limbs were pale and cold to the mid-leg with delayed capillary refill, although no motor or sensory deficits were present. Femoral pulsations were palpable bilaterally, but no Doppler signals were detected in the feet. The electrocardiogram was normal. Accordingly, the patient was kept nil by mouth, and intravenous heparin was initiated.

**Figure 1:**
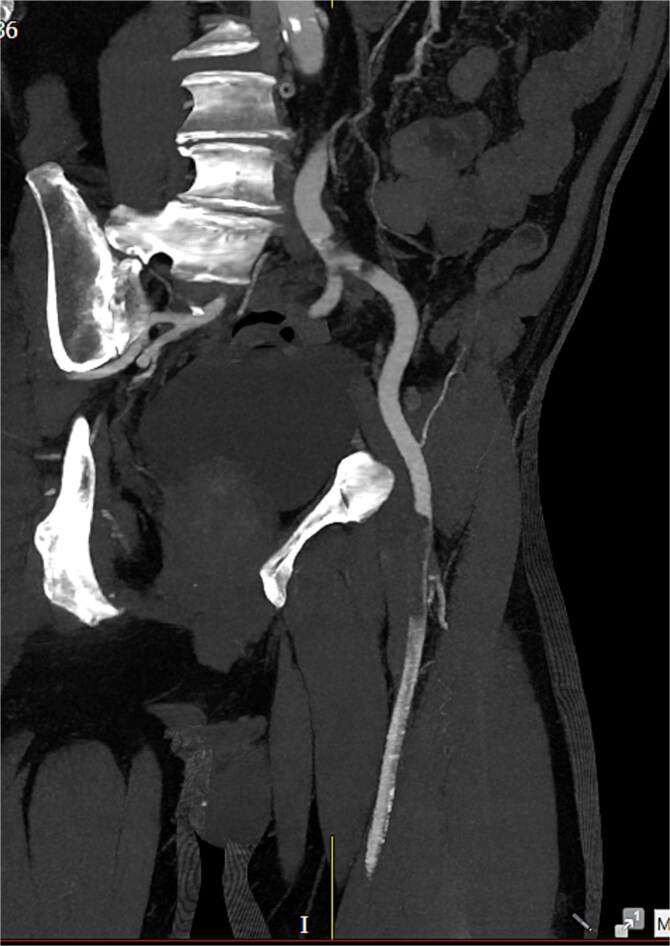
A near-occlusive filling defect at the left common iliac bifurcation. Further embolic material extended into the left common femoral bifurcation, profunda femoris, and superficial femoral artery.

**Figure 2:**
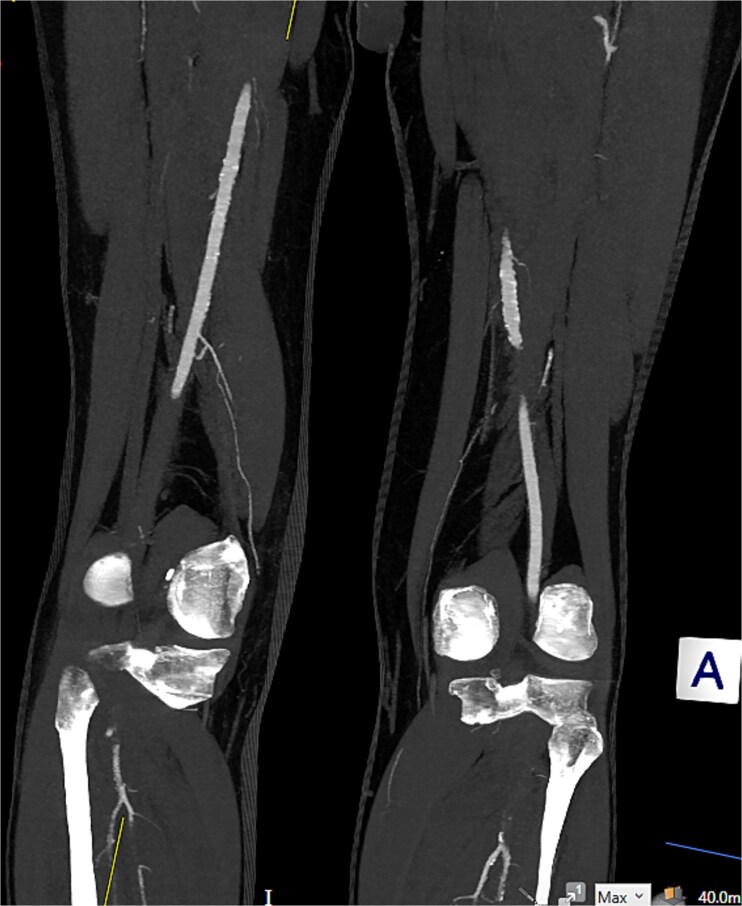
Embolic occlusion of the below-knee popliteal artery extending to the trifurcation, with partial reconstitution of the calf vessels. Emboli are also present in the posterior tibial and peroneal arteries.

Computed tomography (CT) angiography (Figs 1 and 2) revealed a near-occlusive filling defect at the left common iliac bifurcation. Further embolic material extended into the left common femoral bifurcation, profunda femoris, and superficial femoral artery. An embolic occlusion was also present in the below-knee popliteal artery, extending to the trifurcation, with partial reconstitution of the calf vessels. Emboli were seen in the posterior tibial and peroneal arteries. On the right side, embolic material was noted in the distal profunda and popliteal artery, with further emboli in the tibio-peroneal trunk. The patient underwent bilateral femoral thromboembolectomy.

Post-operatively, good Doppler signals were detected in the posterior and anterior tibial arteries bilaterally. Thrombus material was sent for microbiological and histological analysis. The patient was maintained on a heparin infusion. An echocardiogram and a CT angiography of the ascending, arch, and descending thoracic aorta were requested to identify the source of the emboli.

**Figure 3:**
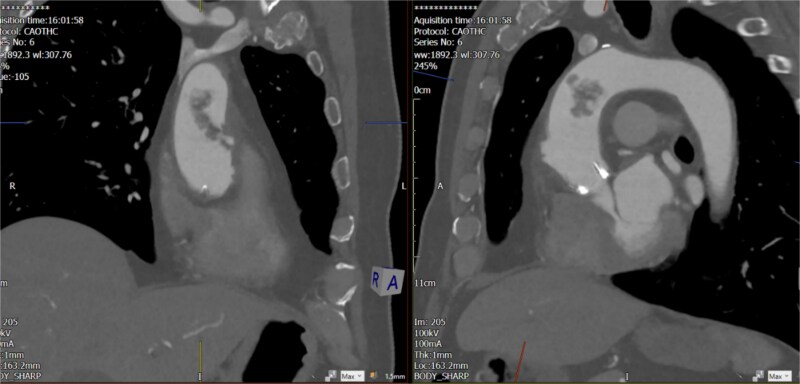
Unstable focal ulceration (1.3 cm) with a 3.5 cm intraluminal filling defect in the ascending aorta. Additional small ulcerations and periaortic soft tissue cuffing are present, along with anterior mediastinal stranding indicative of aortitis.

**Figure 4:**
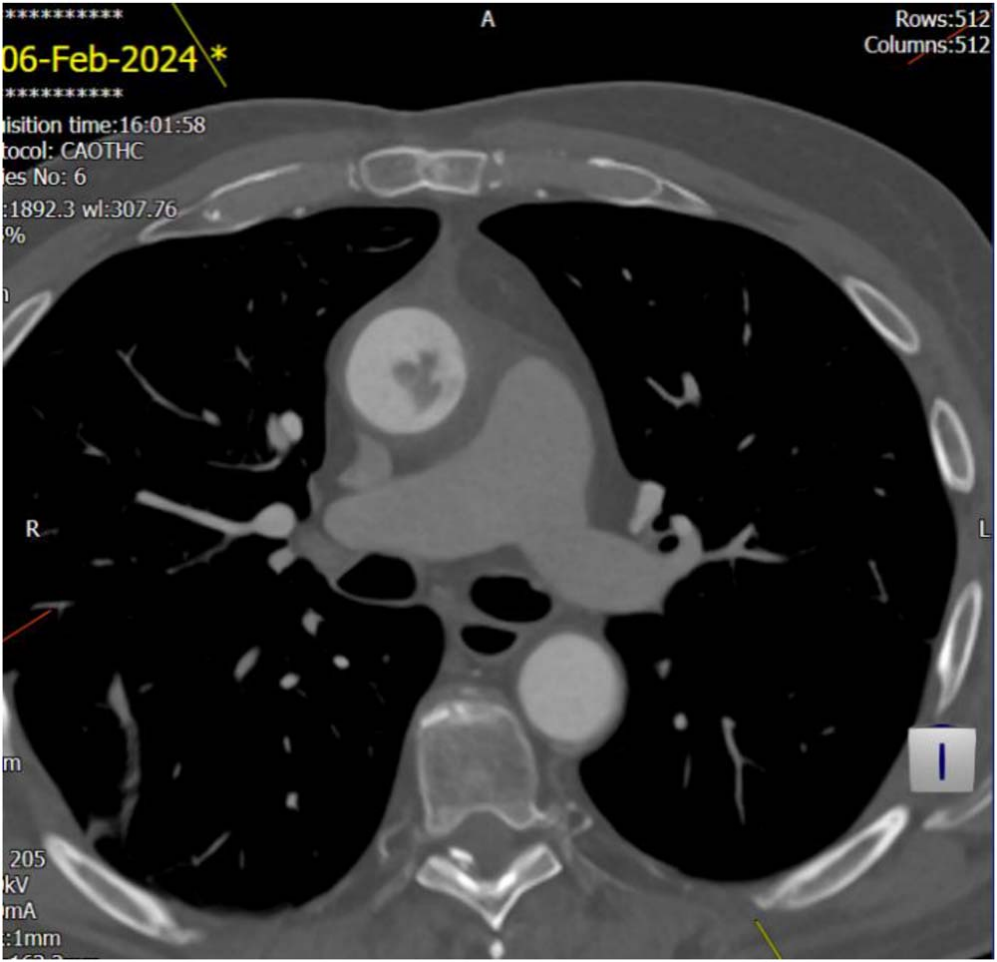
Unstable focal ulceration (1.3 cm) with a 3.5 cm intraluminal filling defect in the ascending aorta. Additional small ulcerations and periaortic soft tissue cuffing are present, along with anterior mediastinal stranding indicative of aortitis.

The echocardiogram showed no valve vegetation or mural thrombus. However, CT angiography of the thoracic aorta (Figs 3 and 4) revealed focal ulceration in the ascending aorta (1.3 cm) with an associated intraluminal filling defect (3.5 cm), appearing unstable. Additional small ulcerations and soft tissue were cuffing around the ascending aorta, with anterior mediastinal stranding suggestive of aortitis.

Histology showed fibrin and degenerate blood, consistent with thrombus. Numerous septate fungal hyphae, some non-branched and others showing acute angle branching, were identified. This suggested intravascular mycosis/fungal infection. Microbiological cultures confirmed *Aspergillus niger*, with a repeat culture and positive serum Beta-D-Glucan and Aspergillus galactomannan antigens. However, blood cultures were negative.

The patient was started on intravenous voriconazole and liposomal amphotericin B (Ambisome), both later switched to oral formulations. Broad-spectrum antibiotics were also administered. During the admission, the patient developed acute kidney injury due to a thrombus in the inferior branch of the left renal artery, as confirmed by repeat CT angiography.

On day thirteen, the patient experienced drooling, confusion, and dysarthria. CT head showed no acute intracranial pathology. The stroke team diagnosed it as a transient ischemic attack, and the symptoms resolved completely.

Importantly, these embolic events continued to occur despite appropriate medical management with intravenous heparin infusion and antifungal therapy, highlighting the limitations of pharmacological treatment alone.

A repeat CT angiography of the entire aorta demonstrated that the floating thrombus in the ascending aorta remained largely unchanged in both size and morphology, suggesting persistent instability and a high risk of further embolization.

This lack of response underscored the need for a more definitive intervention. Consequently, in light of the failure of medical therapy, the decision was made to pursue high-risk surgical intervention through a re-do sternotomy for removal of the infected thrombus from the ascending aorta. The estimated mortality risk was over 60%.

Surgery was successfully performed, and the thrombus was sent for microbiological analysis. The previously replaced tissue valve was intact and was not replaced.

The microbiology culture of the excised thrombus revealed heavy growth of *Enterococcus faecalis* and moderate growth of *Aspergillus* species. Consequently, the patient was prescribed lifelong extended antibiotics and antifungal therapy. He was also discharged on Apixaban 5 mg twice daily and aspirin 75 mg twice daily.

The patient was regularly followed up in vascular and cardiothoracic outpatient clinics. At his most recent review, 8 months post-operation, he was recovering well and gradually resuming his normal life.

## Discussion

*Aspergillus niger* is a species of filamentous fungus commonly found in the environment, particularly in soil, decaying vegetation, air, and indoor environments. It is a member of the *Aspergillus* genus, which includes several species known to cause opportunistic infections in humans [3]. It accounts for ⁓5%–15% of all *Aspergillus*-related infections, with a higher prevalence in otomycosis (up to 90%) and a minor role in invasive aspergillosis, particularly among immunocompromised individuals. The overall incidence of invasive aspergillosis in high-risk populations ranges from 1% to 15%, though *Aspergillus niger* represents a small subset of these cases [4, 5].

In a comprehensive review, Pasqualotto *et al.* identified over 500 cases of surgical site infections caused by Aspergillus, with cardiac surgery being the most common type of surgical intervention, accounting for 188 cases. The review noted a predominance of cases among males and a particular inclination toward infections of the aortic valve, which was affected in 61% of the patients. The median time from surgery to diagnosis was 2.7 months, and the organism identified in 59% of the cases was *Aspergillus* fumigatus. Only 43% of the patients received an antemortem diagnosis, and positive blood cultures were found in just 6% of cases. Late embolization was reported in several cases and contributed to the diagnosis for 17% of the patients, emphasizing the importance of submitting thrombus samples for microbiological evaluation. The overall mortality rate associated with these infections was a staggering 93%, remaining at 81% even after surgical intervention. In this review, the incidence of *Aspergillus* niger infection was noted to be 11%, although the distinction between endocarditis and aortitis was not made [1].

Though *Aspergillus niger* accounts for a smaller proportion of invasive aspergillosis cases compared to *Aspergillus fumigatus*, its clinical outcomes can be severe due to its angioinvasive potential and resistance patterns to some antifungals [3, 6, 7].

The diagnosis of *Aspergillus niger* infection, particularly in invasive disease, can be challenging and typically requires a combination of clinical suspicion, imaging, microbiological, and histopathological confirmation. The culture of clinical specimens (e.g. thrombus, tissue biopsy, or respiratory samples) remains a cornerstone, with *Aspergillus niger* characterized by black colonies and distinct conidial structures on microscopy. Non-culture-based methods such as serum galactomannan antigen and (1 → 3)-β-D-glucan assays are widely used to support early diagnosis, though they are more validated for *Aspergillus fumigatus*. Histopathological evidence of angioinvasion with septate hyphae, often showing acute-angle branching, supports the diagnosis of invasive aspergillosis. Imaging studies like CT or magnetic resonance imaging can reveal features suggestive of fungal involvement, particularly in the lungs or vasculature. Polymerase chain reaction–based assays and fungal biomarkers are emerging tools for species-specific identification, though their routine clinical use varies [8].

Management of *Aspergillus niger* infections, especially invasive disease, focuses on prompt initiation of systemic antifungal therapy, often combined with surgical intervention in cases of vascular or localized tissue involvement. The first-line antifungal agent recommended for invasive aspergillosis, including infections caused by *Aspergillus niger*, is voriconazole, due to its proven efficacy, favorable tissue penetration, and fungistatic activity against *Aspergillus* species.

Liposomal amphotericin B is also used, particularly in cases of intolerance to triazoles, refractory infections, or when rapid fungicidal activity is desired. Combination therapy with voriconazole and an echinocandin (e.g. caspofungin) may be considered in select high-risk or critically ill patients, although robust clinical trial data are limited [9, 10].

When *Aspergillus niger* infection involves major blood vessels such as the aorta or large peripheral arteries, the prognosis is generally poor and associated with a high risk of morbidity and mortality. This is due to the fungus’s angioinvasive nature, which can lead to thrombosis, distal embolization, vascular rupture, and organ ischemia. Mortality rates in such cases can exceed 60%–80%, particularly when diagnosis is delayed or when surgical intervention is not feasible [8, 11].

The risk of recurrent embolic events remains high even with appropriate antifungal therapy, as medical management alone is often insufficient to eliminate fungal thrombi within large vessels. Surgical resection or thrombectomy is frequently required to prevent life-threatening complications. The presence of infected thrombus within the aorta or around prosthetic material (e.g. valves or grafts) further complicates management and worsens prognosis, often necessitating lifelong antifungal therapy to prevent relapse [8, 11].

In conclusion, aspergillus niger aortitis is a rare but life-threatening condition that demands immediate attention. Given the high mortality rate, it is imperative to maintain a strong clinical suspicion in patients with a history of cardiac surgery. Prompt initiation of antifungal therapy, along with surgical intervention, are critical to significantly improve survival rates.
